# Evaluation of highly sensitive diagnostic tools for the detection of *P. falciparum* in pregnant women attending antenatal care visits in Colombia

**DOI:** 10.1186/s12884-020-03114-4

**Published:** 2020-07-31

**Authors:** A. M. Vásquez, G. Vélez, A. Medina, E. Serra-Casas, A. Campillo, I. J. Gonzalez, S. C. Murphy, A. M. Seilie, X. C. Ding, A. Tobón Castaño

**Affiliations:** 1 Grupo Malaria, Facultad de Medicina, Universidad de Antioquia, Carrera 53 No. 61–30, Lab 610, Medellín, Colombia; 2 Independent Consultant, FIND, Geneva, Switzerland; 3 FIND, Geneva, Switzerland; 4 Malaria Molecular Diagnostic Laboratory, Departments of Laboratory Medicine and Microbiology and the Center for Emerging and Re-emerging Infectious Diseases, 750 Republican St, Seattle, WA 98109 USA

**Keywords:** Malaria in pregnancy, Diagnostics, Rapid diagnostic test, Microscopy, Nucleic acid amplification techniques, Loop mediated isothermal DNA amplification (LAMP)

## Abstract

**Background:**

In low transmission settings early diagnosis is the main strategy to reduce adverse outcomes of malaria in pregnancy; however, microscopy and rapid diagnostic tests (RDTs) are inadequate for detecting low-density infections. We studied the performance of the highly sensitive-RDT (hsRDT) and the loop mediated isothermal DNA amplification (LAMP) for the detection of *P. falciparum* in pregnant women.

**Methods:**

A cross-sectional study was conducted in two malaria-endemic municipalities in Colombia. We screened pregnant women in the context of an antenatal care program in health facilities and evaluated five tests (microscopy, conventional RDT, hsRDT, LAMP and nested polymerase chain reaction-PCR) for the detection of *P. falciparum* in peripheral blood, using a quantitative reverse transcription PCR (qRT-PCR) as the reference standard. Diagnostic performance of hsRDT and LAMP were compared with routine testing.

**Results:**

The prevalence of *P. falciparum* was 4.5% by qRT-PCR, half of those infections were subpatent. The sensitivity of the hsRDT (64.1%) was slightly better compared to microscopy and cRDT (59 and 53.8% respectively). LAMP had the highest sensitivity (89.7%) for detecting *P. falciparum* and the ability to detect very low-density infections (minimum parasite density detected 0.08 p/μL).

**Conclusions:**

There is an underestimation of *Plasmodium* spp. infections by tests routinely used in pregnant women attending antenatal care visits. LAMP methodology can be successfully implemented at local hospitals in malaria-endemic areas. The relevance of detecting and treating this sub-patent *P. falciparum* infections in pregnant women should be evaluated.

**Trial registration:**

ClinicalTrials.gov, Identifier: NCT03172221, Date of registration: May 29, 2017.

## Background

Pregnant women are especially susceptible to *Plasmodium* infection and malaria in pregnancy is associated with well-documented adverse outcomes for both the mother and foetus; the main deleterious effects include maternal anemia, miscarriage, premature delivery and low birth weight [1–4]. In the Americas region, more than four million women at reproductive age are at risk of malaria infection each year; of these, 3 million pregnancies are considered at risk of infection with *Plasmodium falciparum* [5]*.* Since the overall transmission intensity in the continent is relatively low, intermittent preventive treatment during pregnancy is not recommended and the main strategies to limit the burden and consequences of malaria are based on “prompt diagnosis and effective treatment” combined with the use of long-lasting insecticide treated bed nets [6, 7]. In Colombia, the current national guidelines for malaria control recommend the active detection of cases at each antenatal care visit in all pregnant women living in endemic areas of the country [6]. However, the diagnosis of malaria in pregnancy by conventional diagnostic tools, such as microscopy or rapid diagnostic tests (RDTs), remains challenging for the detection of low-density infections, common in areas of low to moderate endemicity such as Latin America [4, 7, 8]. Moreover, the unique ability of *P. falciparum* parasites to massively sequester in the placenta also contributes to a reduced detectability of maternal infections in peripheral blood [4, 8, 9].

Although microscopy remains the mainstay of malaria diagnosis in many endemic settings, this method provides limited sensitivity and requires well-trained personnel as well as adequate laboratory reagents and equipment. In Colombia, microscopy-based diagnosis has been shown to miss between 20 and 75% of maternal infections detected in peripheral blood by Polymerase Chain Reaction (PCR) [10–15]. RDTs are inexpensive and can be used by minimally trained health workers, offering therefore a useful alternative to microscopy [8, 16–20]. However, evidence indicate that RDT performance may be suboptimal for the detection of maternal infections, especially among asymptomatic women [17–20]; likewise, the few studies that have evaluated RDT performance in Colombia suggest that this point-of-care tool does not provide significantly improved sensitivity as compared to microscopy, i.e. failure to detect half of the maternal infections in peripheral blood from asymptomatic pregnant women [14].

More sensitive diagnostic tools are needed for an accurate identification of infections. Molecular methods based on nucleic acid amplification techniques, such as PCR, can detect very low parasite densities, but are generally impractical for wide-scale clinical use as they rely on sophisticated equipment and highly-skilled staff, which are rarely available in most malaria endemic settings [21, 22]. Many efforts have been made to develop molecular test suitable for field application in remote areas, such as the loop-mediated isothermal DNA amplification (LAMP). The LAMP assays are relatively simple to implement, as they require minimal laboratory infrastructure and allow assessing the results by visual observation of the reaction [23]. LAMP-based malaria diagnosis has been shown to provide performance comparable to PCR performed in a reference laboratory for the detection of malaria infections in pregnant women from both high-transmission (Ethiopia) [23] and low-transmission (Colombia) settings [14].

A malaria RDT with a significantly improved sensitivity for the detection of *P. falciparum* histidine rich protein 2 (HRP2) is available since 2017 (Alere™ Malaria Ag Pf ultra-sensitive RDT). This “highly sensitive” RDT (hsRDT) showed an improved sensitivity as compared to conventional RDTs (cRDT) when using PCR as reference standard in samples collected from asymptomatic individuals, suggesting that the new test could be a useful tool for malaria elimination strategies [24–26]. In field conditions, the hsRDT performed consistently better than cRDT and microscopy in asymptomatic individuals from Myanmar and the sensitivity was at least two times better (51.4% versus 25.2%) [25].

By combining a significantly improved sensitivity together with the ease-of-use and affordability of cRDT, the hsRDT represents a potentially useful tool to identify the especially hard-to-detect malaria infections in pregnant women. In a previous retrospective study using peripheral and placental specimens collected in Colombian pregnant women, the hsRDT showed a slightly improved sensitivity (85.7%) for detecting *P. falciparum* infections when compared to microscopy (77.1%) and cRDTs (77.1–82.8%), using nested PCR (nPCR) as reference standard [15]. The difference in test accuracy between the hsRDT and other standards of practice was even more evident in asymptomatic participants (with sensitivity estimates of 71.4% for the hsRDT, 50.0% for microscopy and ranging from 50.0 to 64.3% for cRDTs) [15]. However, this initial study was conducted using stored whole blood samples under laboratory conditions, and with highly trained laboratory technicians, limiting the extrapolation of these results to what can be expected when using the test prospectively on fresh capillary blood samples in a standard clinical context [25]. This study aimed to assess the performance of the hsRDT and LAMP for the detection of *P. falciparum* during routine antenatal care visits in two municipalities of Colombia. In addition, the results were compared to the performance of other standards of practice such as the microscopy or the cRDTs.

## Methods

### Study design

A cross-sectional study was conducted in two endemic municipalities from Colombia, between August 2017 and January 2018. The two municipalities are considered areas of high risk of infection, as defined by annual parasite incidence (number of cases per 1000 inhabitants) of 100.8 in Quibdó (2016) and 16 in Tumaco (2016). *P. falciparum* is the predominant species in both sites, representing 70 and 90% of all malaria cases, respectively [27]. It is estimated that at least half a million women at reproductive age live in the two malaria endemic Departments where the study took place (Nariño and Chocó) [28].

In Colombia, *P. falciparum* parasites mutant for the pfhrp2 gene have been reported in the Amazonas Department (38.5%, 15/39), southern Colombia. These parasites were also mutant for the pfhrp3 gene, which make them undetectable by HRP2-based malaria RDTs. However, the prevalence of this genetic deletions is rare in other endemic regions of Colombia, including where this study was conducted [29].

### Study population

Pregnant women, self-presenting at health centers for antenatal care visits and who met the following inclusion criteria were included: confirmed pregnancy, registered to the antenatal care program in the local health center, ≥15 years old and resident for at least 1 year in the study site. The following exclusion criteria were considered: past history of malaria or antimalarial drugs in the last 3 months, having tested positive for malaria by microscopy or cRDT in any previous study visit, symptoms and signs of severe malaria as defined by WHO. Malaria-infected pregnant women were treated based on results of diagnostic tests accepted locally – i.e. microscopy and cRDT – and according to local policy.

The sample size was calculated according to a previously published methodology [30] to obtain sensitivity and specificity estimates of the index test with 95% confidence intervals (CI) of +/− 10%. Assuming a malaria prevalence of 4.8% in the study area and population, an expected sensitivity and specificity of the hsRDT of 90% when compared to quantitative reverse transcription PCR (qRT-PCR), calculations resulted in a minimal target sample size of 721 study participants.

### Ethics statement

All study participants provided a written informed consent or informed assent (in the case of minor pregnant women, younger than 18 years) to participate in the study. The study protocol and documents were reviewed and approved by the ethics committee of Medical Faculty-Universidad de Antioquia (Record 010 of 22-05-2017). Additional consent was also obtained for future use of biological samples. For women younger than 18 years old, additional written approval from the parent or legal guardian was required. The study was conducted in accordance with the Declaration of Helsinki and local rules and regulations of Colombia.

### Diagnostic test procedures

Each participant provided 290 μL of finger-prick blood that was handled as follows: 10 μL for cRDT and hsRDT (5 μL for each test); 20 μL for microscopy; 60 μL for DNA extraction and LAMP; 100 μL spotted on filter papers (Whatman 3) for DNA extraction and nPCR; and 100 μL spotted on filter papers (Whatman 903) for RNA extraction and qRT-PCR. Testing by microscopy, cRDT (Malaria Ag P.f/P.v, Abbott Diagnostics, 05FK80), hsRDT (Alere Malaria Ag P.f. Ultra-Sensitive, Abbott Diagnostics, 05FK140) and LAMP (Loopamp™ MALARIA Kit, Eiken Chemical Company) were performed in field conditions in the local hospitals.

Testing by microscopy, cRDT (SD BIOLINE Malaria Ag P.f. P.v, Abbott Diagnostics, 05FK80, lot number 05DDB028A), hsRDT (Alere Malaria Ag P.f. Ultra-Sensitive, Abbott Diagnostics, 05FK140, lot number 05LDB005A) and LAMP (Loopamp™ MALARIA Pan/Pf Detection Kit, Eiken Chemical Company) were performed in field conditions and conducted in the public facilities of local hospitals. The cRDT and hsRDT were performed by community health workers, microscopy by a malaria microscopist and LAMP by a clinical microbiologist. The study staff received appropriate training before the study initiation.

The hsRDT and cRDT were performed according to the manufacturer’s instructions. The test results were read at 15 min for cRDT by one independent reader and at 20 min for hsRDT by two independent readers blinded to each other. The results were interpreted as invalid (no control line or red background), positive (control line and test line), or negative (control line, no test line).

Microscopy was performed according to the WHO guidelines [31]. A smear was considered negative if no asexual parasites were found after examination of 200 high power fields. For positive smears, parasite density was calculated by counting asexual parasites per 500 leukocytes. Each smear was read by a second microscopist who was blinded to the hsRDT and cRDT as well as the microscopy result of the first reader. Discordant results were resolved by a third microscopist according to the Obare Method calculator [32].

For LAMP, DNA extraction was performed using the boil and spin method as described elsewhere [33]. A combination of a *Plasmodium spp* (Pan-LAMP) and a *P. falciparum* specific (Pf-LAMP) LAMP assays amplifying *Plasmodium* mitochondrial DNA were used according to the manufacturer’s instructions. All Pan-LAMP positive samples were subsequently tested using Pf-LAMP. Results were read by visual fluorescence and classified as negative (Pan-LAMP negative), *P. falciparum* positive (Pan-LAMP and Pf-LAMP positive) or Non-*falciparum* positive (Pan-LAMP positive and Pf-LAMP negative).

A nPCR, based on the amplification of the 18S rRNA gene, was performed as previously described [34]. Briefly, it consists in a first *Plasmodium spp* amplification followed by a nested species-specific PCR (for *P. falciparum, P. vivax* and *P. malarie*). DNA was extracted from blood-spot filter using QIAamp DNA Mini Kit (Qiagen, Ref 51,306), according to the manufacturer’s instructions. The limit of detection of the nPCR was estimated at approximately 1 p/μL.

A qRT-PCR based on the amplification of *Plasmodium* 18S rRNA, was performed at the University of Washington as previously described and was used as reference [35, 36]. Briefly, desiccated dried blood spots (50 μL of blood) were excised from the original cards by contact-free laser cutting to prevent cross-contamination [35] and then nucleic acid extraction on an Abbott m2000sp and qRT-PCR on an Abbott m2000rt was performed as previously described [36]. Quantification was determined against a standard curve of Armored RNA (Asuragen) corresponding to full-length A-type *P. falciparum* 18S rRNA added to parasite-free whole blood. Copies/mL of whole blood was used to estimate p/μL of whole blood using a conversion factor (7.4 × 10^3^ copies per ring-stage *P. falciparum* parasite [36]. This approach allowed sensitive identification of *Plasmodium spp.* infections (limit of detection 0.02 p/μL) and classification as *P. falciparum* or non-*P. falciparum* infections.

### Definitions

Febrile women were those presenting fever (≥37.5 °C axillary temperature) at enrolment or with a self-reported history of fever within the 3 days preceding enrolment [15]. Afebrile women were those without fever or history of fever during the 3 days preceding enrolment. Subpatent infections were defined as positive by qRT-PCR and negative by microscopy and cRDT. Patent infections were defined as those positive by qRT-PCR and by microscopy or cRDT.

### Data processing and analysis

Demographic, clinical, obstetrical information and test outcome was collected using case report form and double entered in Open Clinica system. Descriptive analysis was carried out using SPSS version 25 software and Excel (Microsoft). Test performance analyses were conducted with STATA version 11 software (StataCorp LP, College Station, TX, USA). The Mann-Whitney test was used for comparison of mean parasite densities. Graphs were prepared with Graphpad Prism 8.1. (GraphPad Software, Inc., CA, US), except for the Venn diagrams that were generated with the InteractiVenn tool [37].

## Results

### Demographic and clinical characteristics of the study population

**Fig. 1:**
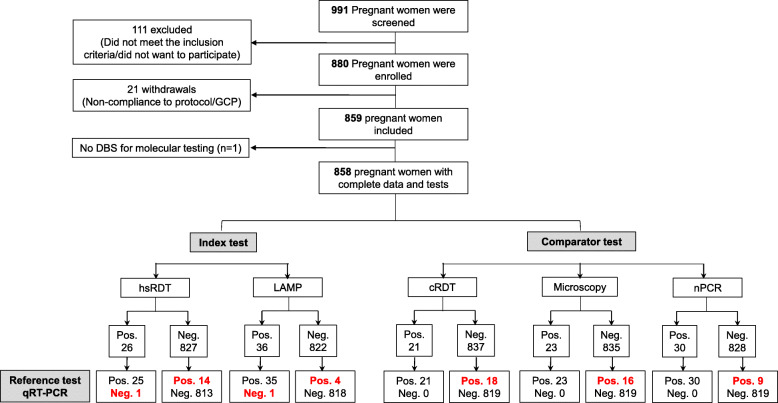
Study participant flow and testing results for *P. falciparum*. The chart shows the total number of pregnant women recruited, as well as the overall number of *P. falciparum* infections detected by each test. Red and bold text: Discrepant results when compared with the reference test. Pos. (positive); Neg. (negative); LM (Light Microscopy); cRDT (conventional Rapid Diagnostic Test); hsRDT (highly sensitive Rapid Diagnostic Test); nPCR (nested Polymerase Chain reaction), LAMP (Loop-mediated isothermal amplification), qRT-PCR (Quantitative Reverse Transcription polymerase chain reaction)

A total of 991 pregnant women were screened and 880 (89%) were enrolled (Fig. 1). Of those, 21 study participants were excluded from the analysis due to non-compliance with the study protocol (minors that assented to participate with the consent of adult care givers instead of legal parents) and one due to missed dried blood spot for molecular analysis. A total of 858 study participants (98% of the enrolled participants) had complete data for microscopy, hsRDT, cRDT, LAMP, nPCR and qRT-PCR, and were therefore included in the analysis. The median age (IQR) of participants was 24 years (20–29) and 30.5% were primigravidae. The median gestational age at enrollment was 22 weeks (range: 15–30 weeks) and approximately one-fifth of the participants (179/858, 21%) were enrolled during the first trimester. Other baseline characteristics are shown in Table 1.

**Table 1:** Baseline characteristics of study participants at enrolment

	Total (***N*** = 858)
**Age (years):** median (IQR)	24 (20–29)
**≤18 years old: n (%):** n (%)	97 (11.3)
**Gravidity (pregnancies):** median (IQR)	2 (1–3)
**Primigravida:** n (%)	262 (30.5)
**Gestational age (weeks):** median (IQR)	22 (15–30)
**First trimester:** n (%)	179 (20.9)
**Axillary temperature (°C):** median (IQR)^a^	36.5 (36.3–36.7)
**Fever at enrollment:** n (%) ^a^	7 (0.8)
**History of fever (72 h):** n (%)	85 (9.9)
**Febrile:** n (%) ^a^	88 (10.3)
**Municipality:** n (%)	
Quibdó	394 (45.9)
Tumaco	464 (54.1)
**Address type:**	
Rural	182 (21.2)
Urban	676 (78.8)

^a^1 missing; *IQR* Interquartile range

### Parasitological characteristics of the study population

A total of 47 out of 858 (5.5%) *Plasmodium* infections were detected by qRT-PCR (Table 2), including 38 (81%) *P. falciparum,* 8 (17%) non-*P. falciparum* and 1 (2%) mixed infection. Subpatent infections (*n* = 24) represented 42% (16/38) of *P. falciparum* infections and 100% (8/8) of non-*P. falciparum* ones.

**Table 2:** *Plasmodium* spp. positivity rate by diagnostic test in peripheral blood samples collected from pregnant women

Positive test: n (%) [***N*** = 858]	***Plasmodium*** spp***.***	Species-specific			Non-***P.f.***
		***P. falciparum***	***P. vivax***	***P. malariae***	
**Microscopy**	23 (2.7%)	23 (2.7%) ^c^	1 (0.1%) ^c^	0 (0%)	N/A
**cRDT**^**a**^	21 (2.4%)	21 (2.4%) ^c^	1 (0.1%) ^c^	N/A	N/A
**hsRDT**^**b**^	N/A	26 (3.0%)	N/A	N/A	N/A
**LAMP**	45 (5.2%)	36 (4.2%)	N/A	N/A	9 (1.0%)
**nPCR**	36 (4.2%)	30 (3.5%) ^c^	4 (0.5%) ^c^	3 (0.3%)	N/A
**qRT-PCR**	47 (5.5%)	39 (4.5%) ^d^	N/A	N/A	9 (1.0%) ^d^

*cRDT* conventional Rapid Diagnostic Test, *hsRDT* highly sensitive Rapid Diagnostic Test, *nPCR* nested Polymerase Chain Reaction, *LAMP* Loop-mediated isothermal amplification, *qRT-PCR* Quantitative Reverse Transcription polymerase chain reaction

^a^Only *P. falciparum* and *P. vivax*. ^b^Only *P. falciparum* detected. Five samples (0.6%) with indeterminate results. ^c^Includes one mixed infection of *P. falciparum* and *P. vivax*. ^d^Includes one mixed infection of *P. falciparum* and non-*P. falciparum* species

Infected pregnant women showed parasite densities ranging from 0.03 to 8145 parasites per microliter (p/μL) with a geometric mean of 13.2 p/μL. The parasite density was significantly lower in subpatent (geo. Mean = 0.38 p/μL) and afebrile (geo. Mea*n* = 9.2 p/μL) infections, as compared to patent (geo. Mean = 159 p/μL, *p* < 0.001) and febrile (geo. Mean = 270 p/μL) infections (Additional file 1). Parasite densities tended to be higher in primigravidae compared to women with one or more previous pregnancies, although the difference was not significant (*p* = 0.217). Likewise, infections detected during the first trimester of pregnancy showed higher densities than those occurring in the second or third trimester, but the difference did not reach statistical significance (*p* = 0.053) (Additional file 1).

### Clinical performance of the index and comparator tests for *P. falciparum* detection

**Fig. 2:**
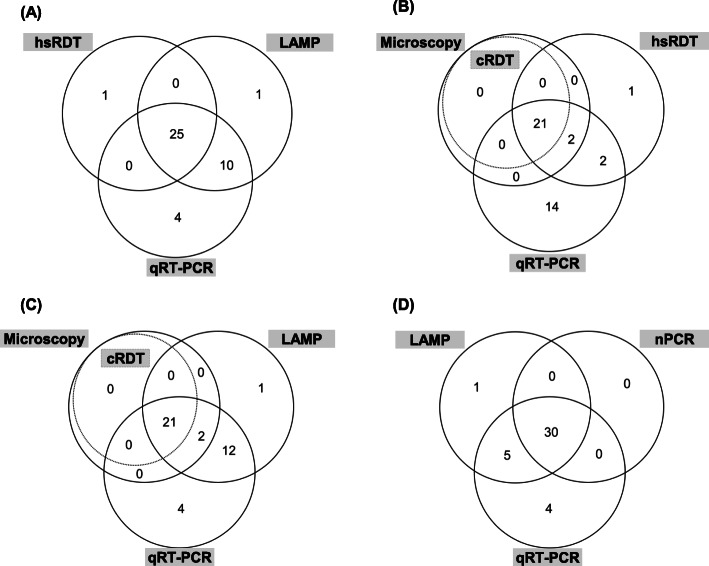
Venn diagram of *P. falciparum* positivity by different diagnostic methods. **a** Positivity by the index tests (hsRDT and LAMP), and the reference test (qRT-PCR); (**b**) & **(c)** Positivity by the standards of practice (LM and cRDT), the reference test (qRT-PCR) and one of the index tests (hsRDT & LAMP, respectively); **(d)** Positivity by the nucleic-acid amplification tests (nPCR, LAMP, qRT-PCR). Testing conducted in 858 pregnant women during antenatal care visits

The results of the index and comparator tests are presented in Figs. 1 and 2, and the resulting sensitivity, specificity, positive and negative predicting values (PPV, NPV, respectively) are reported in Table 3. The hsRDT and LAMP detected respectively 25 and 35 of the 39 *P. falciparum* infections identified by qRT-PCR (Fig. 2a). Out of the 819 *Plasmodium-*negative samples, both index tests reported one false positive result each (not positive by any other method). This translated in 64.1% (95% CI: 47.2–78.8) sensitivity and 99.9% (95% CI: 99.3–100.0) specificity for the hsRDT, and 89.7% (95% CI: 75.8–97.1) sensitivity and 99.9% (95% CI: 99.3–100.0) specificity for LAMP. The microscopy, cRDT, and nPCR comparator tests identified respectively 23, 21, and 30 of the 39 *P. falciparum* infections, resulting in sensitivity of 59% (95 CI: 42.1–74.4), 53.8% (95 CI: 37.2–67.9), and 76.9% (95CI: 60.7–88.9). All the 819 negative samples were correctly identified as such by these tests, resulting in 100% specificity (95 CI, 99.6–100.0) for each of them. The PPV and NPV estimates were > 96% for all tests evaluated in this study. The hsRDT detected 22% (4/18) of the positive samples missed by the cRDT (Fig. 2b). LAMP showed the highest sensitivity of all diagnostic tests evaluated, detecting between 56% (5/9 as compared to nPCR) and 78% (14/18 as compared to cRDT) of all the infections respectively missed by the other tests (Fig. 2c-d).

**Table 3:** Diagnostic test performance for *P. falciparum* detection, compared to a reference standard (qRT-PCR)

		qRT-PCR		Total	value ***(95% CI)***			
		+	–		Sensitivity	Specificity	PPV	NPV
**Microscopy**	+	23	0	23	59.0%	100.0%	100.0%	98.1%
	–	16	819	835	(42.1–74.4)	(99.6–100.0)	(85.2–100.0)	(96.9–98.9)
**cRDT**	+	21	0	21	53.8%	100.0%	100.0%	97.8%
	–	18	819	837	(37.2–69.9)	(99.6–100.0)	(83.9–100.0)	(96.6–98.7)
**hsRDT**^**a**^	+	25	1	26	64.1%	99.9%	96.2%	98.3%
	–	14	813	827	(47.2–78.8)	(99.3–100.0)	(80.4–99.9)	(97.2–99.1)
**LAMP**	+	35	1	36	89.7%	99.9%	97.2%	99.5%
	–	4	818	822	(75.8–97.1)	(99.3–100.0)	(85.5–99.9)	(98.8–99.9)
**nPCR**	+	30	0	30	76.9%	100.0%	100.0%	98.9%
	–	9	819	828	(60.7–88.9)	(99.6–100.0)	(88.4–100.0)	(97.9–99.5)

+ positive, − negative, *PPV* positive predictive value, *NPV* negative predictive value, *CI* confidence interval. ^a^Five samples with indeterminate result (not included). *qRT-PCR* Quantitative Reverse Transcription polymerase chain reaction, *cRDT* conventional rapid diagnostic test, *hsRDT* highly sensitive rapid diagnostic test, *LAMP* loop-mediated isothermal amplification, *nPCR* nested polymerase chain reaction

We tested the superiority of the hsRDT for the detection of *P. falciparum*, when compared with both microscopy and cRDT (margin of 15% difference; 5% significance level). Although the hsRDT had higher sensitivity than microscopy and cRDT, the superiority was not established (Additional file 2). We also tested the equivalence of the hsRDT compared to PCR (margin of 3% difference, 5% significance level). The sensitivity of the hsRDT cannot be considered equivalent to those achieved by LAMP, nPCR or qRT-PCR (Additional file 2).

Multiple stratification of the study population and the related test sensitivities are reported in Additional file 3. Microscopy, hsRDT, LAMP and nPCR had a sensitivity of 100% for detecting *P. falciparum* infections in febrile pregnant women (Additional file 3). Among afebrile participants, microscopy and cRDT missed half of the infections detected by qRT-PCR (sensitivity of 52.9 and 50.0% respectively) and the hsRDT also missed an important proportion of positive afebrile cases (14/34, 41.2%). LAMP showed a sensitivity of 100% to detect infections during the first trimester of pregnancy as well as in primigravidae at all trimester. Likewise, hsRDT provided better performance among primigravidae and first trimester participants (> 85% sensitivity), as compared to the secundi/multigravidae and second or third trimester women.

Overall, all tests were able to detect all infections with parasite density higher than 100 p/μL (Table 4), however, 63% (24/38) of the *P. falciparum* infections had estimated densities < 100 p/μL. The hsRDT performed better than cRDT to detect densities between 10 to 100 p/μL (9/9 [100%] and 6/9 [66.7%], respectively). Microscopy, cRDT and hsRDT performed poorly below 10 p/μL. However, while the lowest parasitemia detected by the standards of practice (microscopy and cRDT) was 13.1 p/μL and 42.3 p/μL, respectively, the hsRDT was able to identify all the infections over 1.8 p/μL. LAMP had the best performance across low density ranges and was able to detect 75% of the infections below 1 p/μL (highest undetected parasitemia was 1.56 p/μL) (Table 4).

**Table 4:** Frequency distribution of estimated parasite densities of *P .falciparum* infections detected by each diagnostic test

Diagnostic test	n	No. of infections detected by parasite density category (% of qRT-PCR-positive) ^**a**^			
		< 1 p/μL	1–10 p/μL	10–100 p/μL	> 100 p/μL
		(***n*** = 12)	(***n*** = 3)	(***n*** = 9)	(***n*** = 14)
**Microscopy**	22	0 (0)	0 (0)	8 (88.9)	14 (100)
**cRDT**	20	0 (0)	0 (0)	6 (66.7)	14 (100)
**hsRDT**	24	1 (8.3)	0 (0)	9 (100)	14(100)
**LAMP**	34	9 (75.0)	2 (66.7)	9 (100)	14 (100)
**nPCR**	29	5 (41.7)	1 (33.3)	9 (100)	14 (100)

^a^Refers to P. falciparum mono-infections (i.e., mixed infection not included. p/μL: estimated parasites per microliter, *qRT-PCR* Quantitative Reverse Transcription polymerase chain reaction, *cRDT* conventional rapid diagnostic test, *hsRDT* highly sensitive rapid diagnostic test, *nPCR* nested polymerase chain reaction, *LAMP* loop-mediated isothermal amplification

**Fig. 3:**
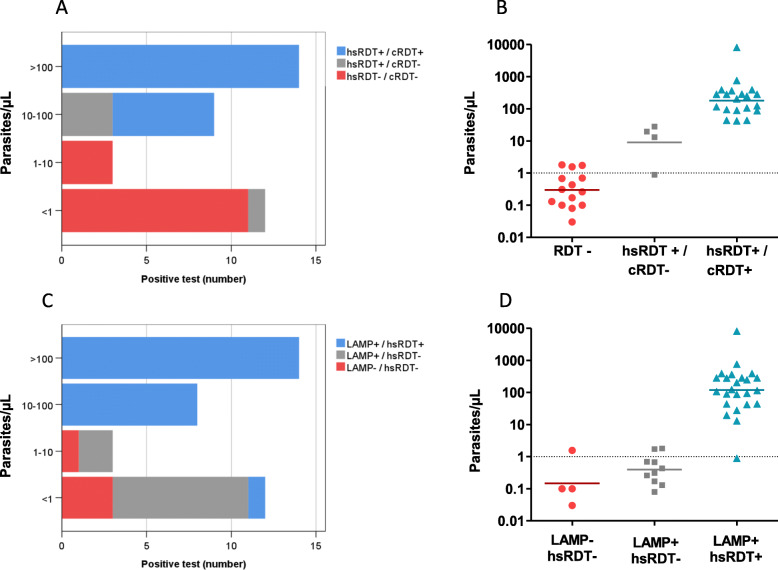
Distribution of *P. falciparum* parasitemias among infected pregnant women, according to positivity by different diagnostic methods. Distribution of parasite densities in 38 samples with *P. falciparum* positivity by qRT-PCR, according to detection by cRDT or hsRDT (**a** and **b**) and by LAMP and hsRDT (**c** and **d**). −, negative; +, positive

Figure 3a shows the positive samples detected by cRDT and hsRDTs, stratified by parasite density. The additional cases detected by the hsRDT but not by cRDT (*n* = 4) ranged from 0.89 to 27.8 p/μL (Fig. 3b). The additional infections detected by LAMP but not by the hsRDT (*n* = 10) ranged from 0.08 to 1.80 p/μL (Fig. 3d).

## Discussion

Here, we screened pregnant women in two Colombian sites in the context of an antenatal care program and evaluated the accuracy of five tests for diagnosing malaria when used in regular clinical settings. Using a highly sensitive qRT-PCR as the reference method (analytical limit of detection 0.02 estimated p/μL of blood), overall results showed that conventional diagnostics tests like microscopy and cRDT were unable to detect a large proportion of asymptomatic and low-density infections. LAMP had the highest sensitivity for detecting *P. falciparum* and hsRDT showed a slightly improved sensitivity as compared to cRDT.

Malaria prevalence in pregnant women was found to be of 5.5% by qRT-PCR, similar to previous studies conducted in Colombia [1, 14, 15], half of those infections were subpatent. As previously reported, cRDTs performed similarly to microscopy to detect *P. falciparum* in pregnant women, and both tests missed a higher number of low-density infections as compared to PCR [14, 17, 19, 20, 38]. This observation highlights the underestimation of actual *Plasmodium* infections by tests routinely used in pregnant women attending antenatal care visits.

The sensitivity of the hsRDT to detect *P. falciparum* infections in peripheral samples from pregnant women (64.1%) was slightly better than that of routinely used tests during antenatal care visits (59% by microscopy and 53.8% by cRDT). Similar findings have been reported in pregnant women [15, 24, 25] and non-pregnant population [24, 25], where the hsRDT sensitivity was higher than cRDT in both, high- and low-transmission settings. The increase in sensitivity achieved by the hsRDT appears to be more significant in afebrile infected individuals that carry low density infections. There was also an increase in sensitivity achieved by the hsRDTs in febrile cases compared to cRDT. The usefulness of this hsRDT in clinical diagnosis was recently evaluated and suggested only in a small proportion of febrile malaria cases the of hsRDT would confer a meaningful advantage over co-RDT [39]. The four *P. falciparum*–positive pregnant women detected by hsRDT but not by cRDT result in a gain of 10% in diagnostic sensitivity, however, the hsRDT was not superior to cRDT. The additional infections detected by hsRDT but not by cRDT had parasite densities ranging from 0.9 to 27.8 p/μL, indicating that hsRDT would confer a meaningful improvement detecting low density infections. The relevance of detecting and treating these additional *P. falciparum* infections in pregnant women should be evaluated [40].

Differences in the sensitivity to detect *P. falciparum* infections were observed among the tests when compared to qRT-PCR as the reference standard. LAMP showing the highest sensitivity (89.7%) and ability to detect very low-density infections (minimum parasite density detected 0.08 p/μL). These results were consistent with previous studies proving higher performance of LAMP for detecting maternal *Plasmodium* infection [14, 23]. In addition, the study also reinforces that LAMP methodology can be successfully implemented at local hospitals in malaria-endemic areas with minimum laboratory infrastructure conditions. Moreover, LAMP has the advantage of being able to detect very low-density infections, *P. falciparum* mutant for the *hrp2/hrp3* genes and non*-P. falciparum* malaria infections.

All the evaluated tests showed an improved performance for first-trimester testing (> 85% sensitivity) compared to second and third trimester, but especially LAMP which was able to detect all infections in the first trimester. This finding is important due to the relevance of early stage detection to prevent deleterious effects of malaria in pregnancy [40]. Overall, a significant proportion of infections (38.5%) remained undetected by cRDT, microscopy, or hsRDT. The clinical relevance of infections detected only by molecular test in pregnant women has been evaluated in some previous studies [41, 42]. Preliminary results from a meta-analysis including 32 studies showed that subpatent malaria infections detected at delivery are associated with decreased maternal haemoglobin levels and possibly low birth weight (AM van Eijk, personal communication). In this sense, LAMP represents a potential tool to detect very low-density infections that may impact the health of the mother and foetus. The relevance of detecting and treating a larger fraction of all *Plasmodium* infections during pregnancy, including low density infections should be studied, as well, the relevance of pregnant women as sentinel population for epidemiological surveillance and the role of pregnant women as transmission reservoir.

LAMP is significantly simpler to implement than some of the more complex molecular methods but still requires several sample processing steps to be conducted and currently retails at a higher cost-per-test than cRDT and hsRDT. The fact that antenatal care is typically provided in clinics where the basic laboratory procedures required by LAMP can be done, together with the fact that there might be a significant direct and long-term clinical benefit in detecting and treating low density *P. falciparum* infections, could make the case for a practical and cost-effective use of LAMP in the frame of integrated maternal and child health services. Yet, further studies are needed to strengthen the relevance of detecting and treating cRDT−/microscopy-negative infections and to guide recommendations on the use of LAMP and hsRDT in pregnant women with inherently low-density *P. falciparum* levels. A validation in different transmission settings and the cost-effectiveness of integrating new tools in the maternal health programs should also be evaluated.

## Conclusions

There is an underestimation of *Plasmodium* spp. infections by tests routinely used in pregnant women attending antenatal care visits, like microscopy and RDTs. The sensitivity of the hsRDT to detect *P. falciparum* infections in peripheral samples was slightly better than that of routine used tests, however, a significant proportion of infections (38.5%) remained undetected by cRDT, microscopy, or hsRDT. LAMP showed the highest sensitivity and ability to detect very low-density infections and their methodology can be successfully implemented at local hospitals in malaria-endemic areas. The relevance of detecting and treating this sub-patent *P. falciparum* infections in pregnant women should be evaluated, to guide recommendations on the use of LAMP and hsRDT.

## Supplementary information

**Additional file 1: Figure S1.** Distribution of *P. falciparum* densities stratified by maternal characteristics. *P. falciparum* mono-infection parasite densities (p/μL; log scale) estimated by RT-qPCR. (A) Distribution of parasite density among 38 qRT-PCR positive pregnant women. (B-E) Mean of parasite density stratified by maternal characteristics. Horizontal bar indicates the geometrical mean. Subpatent: Infection detected by qRT-PCR, but not detected by LM or cRDT. Febrile: Fever at enrolment or reported fever on the last 3 days (1 missing value).

**Additional file 2: Figure S2.** Superiority and equivalence of the hsRDT for the detection of *P. falciparum* during pregnancy compared to other diagnostic tests. A) Superiority testing between hsRDT and Microscopy/cRDT*.* Confidence Intervals (CI) of the difference between sensitivities compared to a superiority margin of + 15%. *One-sided comparative test not significant: *p*-value> 0.05. *Dot: difference; bar: confidence interval; blue: area above the superiority margin.* B) Equivalence testing between hsRDT, LAMP and nPCR. CIs of the difference between sensitivities compared to an equivalence margin of ±3%. * Two one-sided test (TOST) for equivalence not significant: p-value> 0.05. *Dot: difference; thick bar: 90% CI; thin bar: 95% CI; green: area within the equivalence interval. cRDT (conventional Rapid Diagnostic Test); hsRDT (highly sensitive Rapid Diagnostic Test); nPCR (nested Polymerase Chain reaction), LAMP (Loop-mediated isothermal amplification), qRT-PCR (Quantitative Reverse Transcription polymerase chain reaction).*

**Additional file 3: Table S1.** Comparison of diagnostic test performance according to febrile status, maternal age, gravidity and pregnancy trimester of the study participants.

## Data Availability

All data generated or analyzed during this study are included in this published article [and its additional information files]. The datasets used and/or analyzed during the current study are available from the corresponding author on reasonable request.

## Abbreviations

CI: Confidence interval
DNA: Deoxyribonucleic acid
HRP2: Histidine rich protein 2
IQR: (Interquartile range)
LAMP: Loop-mediated isothermal amplification
NPV: Negative predictive value
p/μL: Parasite/μL
*P. falciparum*: *Plasmodium falciparum*
*P. malariae*: *Plasmodium malariae*
*P. vivax*: *Plasmodium vivax*
PCR: Polymerase chain reaction
nPCR: Nested polymerase chain reaction
RDT: Rapid diagnostic test
rRNA: Ribosomal Ribonucleic acid
*spp*: Species
PPV: Positive predictive value
WHO: World health organization
