# Supplementary figures and images for: Evaluation of highly sensitive diagnostic tools for the detection of P. falciparum in pregnant women attending antenatal care visits in Colombia
Source: BMC Pregnancy Childbirth. 2020 Jul 31;20:440. doi: 10.1186/s12884-020-03114-4 (PMC7393871; doi:10.1186/s12884-020-03114-4)

## Slide 1
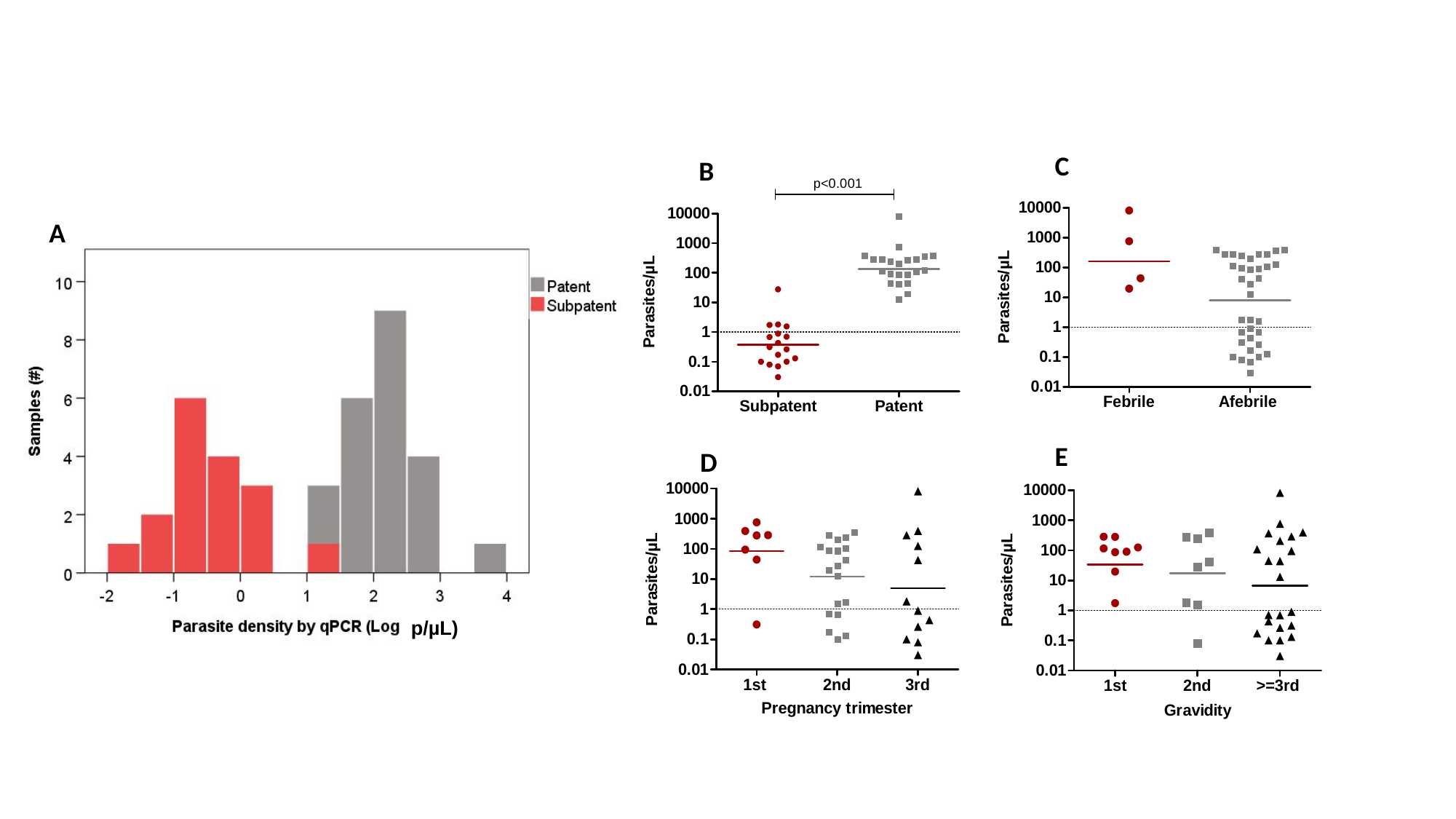

C
B
A
E
D
p/µL)

Supplement: Supplementary file 1 — Additional file 1: Figure S1. Distribution of P. falciparum densities stratified by maternal characteristics. P. falciparum mono-infection parasite densities (p/μL; log scale) estimated by RT-qPCR. (A) Distribution of parasite density among 38 qRT-PCR positive pregnant women. (B-E) Mean of parasite density stratified by maternal characteristics. Horizontal bar indicates the geometrical mean. Subpatent: Infection detected by qRT-PCR, but not detected by LM or cRDT. Febrile: Fever at enrolment or reported fever on the last 3 days (1 missing value). [file 12884_2020_3114_MOESM1_ESM.pptx]
