# Supplementary material for: Evaluation of highly sensitive diagnostic tools for the detection of P. falciparum in pregnant women attending antenatal care visits in Colombia
Source: BMC Pregnancy Childbirth. 2020 Jul 31;20:440. doi: 10.1186/s12884-020-03114-4 (PMC7393871; doi:10.1186/s12884-020-03114-4)
Supplement: Supplementary file 2 — Additional file 2: Figure S2. Superiority and equivalence of the hsRDT for the detection of P. falciparum during pregnancy compared to other diagnostic tests. A) Superiority testing between hsRDT and Microscopy/cRDT. Confidence Intervals (CI) of the difference between sensitivities compared to a superiority margin of + 15%. *One-sided comparative test not significant: p-value> 0.05. Dot: difference; bar: confidence interval; blue: area above the superiority margin. B) Equivalence testing between hsRDT, LAMP and nPCR. CIs of the difference between sensitivities compared to an equivalence margin of ±3%. * Two one-sided test (TOST) for equivalence not significant: p-value> 0.05. Dot: difference; thick bar: 90% CI; thin bar: 95% CI; green: area within the equivalence interval. cRDT (conventional Rapid Diagnostic Test); hsRDT (highly sensitive Rapid Diagnostic Test); nPCR (nested Polymerase Chain reaction), LAMP (Loop-mediated isothermal amplification), qRT-PCR (Quantitative Reverse Transcription polymerase chain reaction). [file 12884_2020_3114_MOESM2_ESM.pptx]

## Slide 1
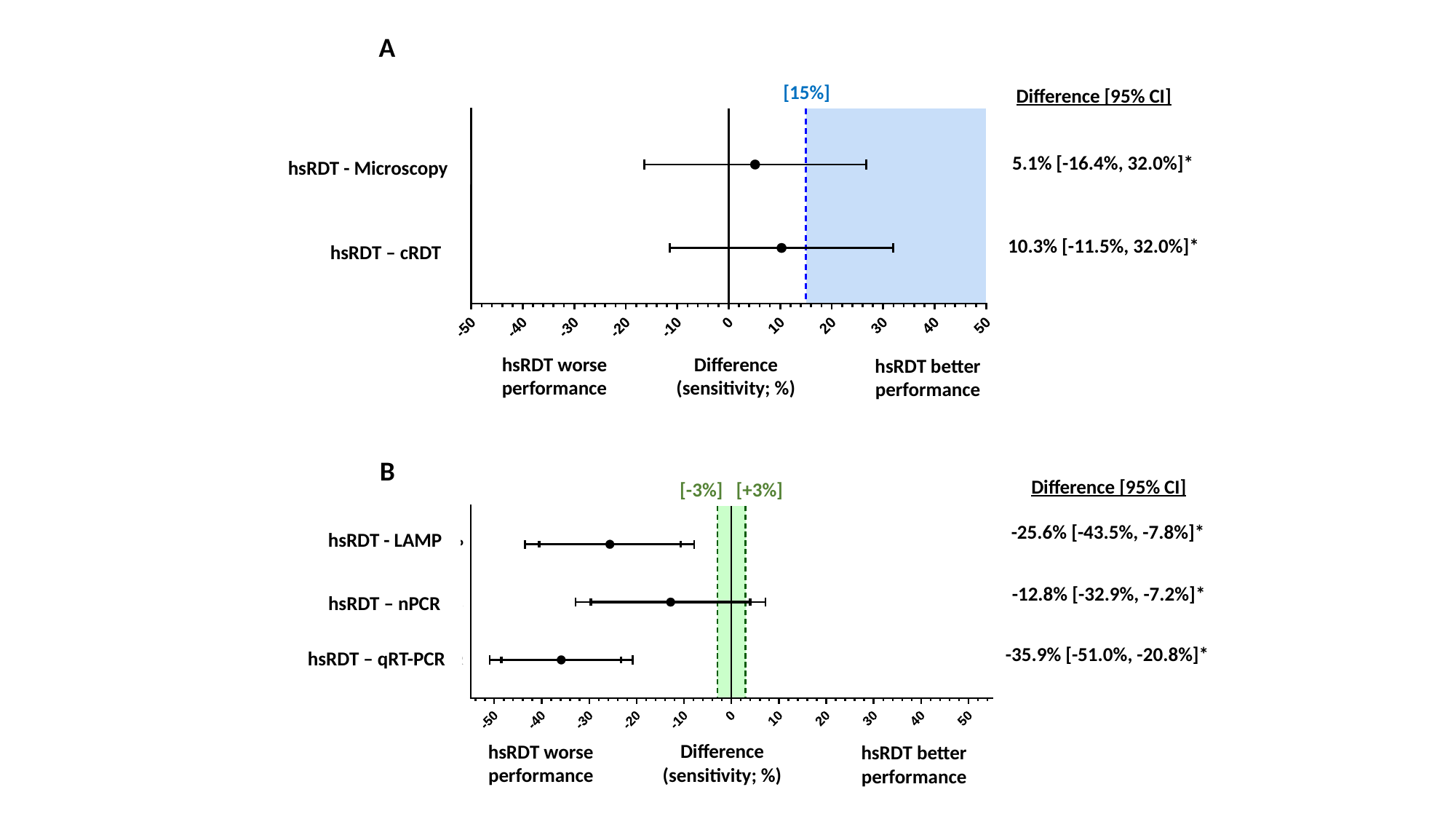

A
[15%]
Difference [95% CI]
5.1% [-16.4%, 32.0%]*
hsRDT - Microscopy
10.3% [-11.5%, 32.0%]*
hsRDT – cRDT
Difference (sensitivity; %)
hsRDT worse performance
hsRDT better performance
Difference [95% CI]
-25.6% [-43.5%, -7.8%]*
-12.8% [-32.9%, -7.2%]*
-35.9% [-51.0%, -20.8%]*
[-3%] [+3%]
hsRDT - LAMP
hsRDT – nPCR
hsRDT – qRT-PCR
Difference (sensitivity; %)
hsRDT worse performance
hsRDT better performance
B
