# Supplementary material for: Evaluation of highly sensitive diagnostic tools for the detection of P. falciparum in pregnant women attending antenatal care visits in Colombia
Source: BMC Pregnancy Childbirth. 2020 Jul 31;20:440. doi: 10.1186/s12884-020-03114-4 (PMC7393871; doi:10.1186/s12884-020-03114-4)
Supplement: Supplementary file 3 — Additional file 3: Table S1. Comparison of diagnostic test performance according to febrile status, maternal age, gravidity and pregnancy trimester of the study participants. [file 12884_2020_3114_MOESM3_ESM.docx]

**ADDITIONAL MATERIAL**

**Additional table 1. Comparison of diagnostic test performance according to febrile status, maternal age, gravidity and pregnancy trimester of the study participants.**

| **Maternal characteristics** | **Sensitivity (95% CI)** | | | | |
| --- | --- | --- | --- | --- | --- |
|  | **Microscopy** | **cRDT** | **hsRDT** | **LAMP** | **nPCR** |
| **Febrile status** |  |  |  |  |  |
| Not febrile (n=769^) | 52.9% (35.1 - 70.2) | 50.0% (32.4 - 67.6) | 58.8% (40.7 - 75.4) | 88.2% (72.5 - 96.7) | 73.5% (55.6 - 87.1) |
| Febrile (n=88) | 100.0% (47.8 - 100.0) | 80.0% (28.4 - 99.5) | 100.0% (47.8 - 100.0) | 100.0% (47.8 - 100.0) | 100.0% (47.8 - 100.0) |
| P value * | 0.066 | 0.349 | 0.139 | 1 | 0.318 |
| **Maternal age** |  |  |  |  |  |
| ≤18y (n=97) | 55.6% (21.2 - 86.3) | 44.4% (13.7 - 78.8) | 66.7% (29.9 - 92.5) | 100.0% (66.4 - 100.0) | 77.8% (40.0 - 97.2) |
| >18y (n=761) | 60.0% (40.6 - 77.3) | 56.7% (37.4 - 74.5) | 63.3% (43.9 - 80.1) | 86.7% (69.3 - 96.2) | 76.7% (57.7 - 90.1) |
| P value * | 1 | 0.706 | 1 | 0.556 | 1 |
| **Previous pregnancies** |  |  |  |  |  |
| Primigravida (n=262) | 87.5% (47.3 - 99.7) | 75.0% (34.9 - 96.8) | 87.5% (47.3 - 99.7) | 100.0% (63.1 - 100.0) | 87.5% (47.3 - 99.7) |
| ≥1 preg. (n=596) | 51.6% (33.1 - 69.8) | 48.4% (30.2 - 66.9) | 58.1% (39.1 - 75.5) | 87.1% (70.2 - 96.4) | 74.2% (55.4 - 88.1) |
| P value * | 0.109 | 0.247 | 0.218 | 0.563 | 0.653 |
| **Pregnancy trimester** |  |  |  |  |  |
| 1st (n=179) | 85.7% (42.1 - 99.6) | 85.7% (42.1 - 99.6) | 85.7% (42.1 - 99.6) | 100.0% (59.0 - 100.0) | 100.0% (59.0 - 100.0) |
| 2nd/3rd (n=679) | 53.1% (34.7 - 70.9) | 46.9% (29.1 - 65.3) | 59.4% (40.6 - 76.3) | 87.5% (71.0 - 96.5) | 71.9% (53.3 - 86.3) |
| P value * | 0.206 | 0.098 | 0.386 | 1 | 0.169 |

*ˆOne missing data*, * *Fisher’s exact test, CI (confidence Interval).*

In all cases the specificity of tests was high ranging from 99.6 to 100%.
